# Extraction, Characterization, and Molecular Weight Determination of* Senna tora* (L.) Seed Polysaccharide

**DOI:** 10.1155/2015/928679

**Published:** 2015-11-12

**Authors:** Harshal A. Pawar, K. G. Lalitha

**Affiliations:** ^1^Department of Quality Assurance, Dr. L. H. Hiranandani College of Pharmacy, Ulhasnagar, Maharashtra 421003, India; ^2^Department of Pharmaceutical Chemistry, Ultra College of Pharmacy, No. 4/235, College Road, Thasildar Nagar, Madurai, Tamil Nadu 625020, India

## Abstract

The objective of the present work was extraction of polysaccharide from* Senna tora* L. seed and its characterization as a pharmaceutical excipient. Polysaccharide extraction was based on mechanical separation of the endosperm of seeds of* Senna tora*, water dissolution, centrifugation, and precipitation with acetone. Standard procedures were used to study the viscosity, micromeritic properties, and microbial bioburden. Accelerated stability study was carried out on isolated polysaccharide for six months at 40°C/75 RH as per ICH guidelines. The gum obtained from* S. tora* seeds was an amorphous free flowing odourless powder with dull brown colour (yield = 35% w/w). The bulk density, tapped density, and angle of repose data reveal that* S. tora* gum possesses good flow property. The intrinsic viscosity obtained was 1.568 dL/g. The average molecular weight of purified* S. tora* gum was found to be 198 kDa by intrinsic viscosity method. The results indicated that viscosity of gum solution increases with increase in temperature. FTIR study revealed the absence of degradation or decomposition of polysaccharide at accelerated stability conditions for six months. It has been concluded that extracted polysaccharide can be used as pharmaceutical excipient in terms of flow behavior, microbial properties, and stability.

## 1. Introduction

The pharmaceutical applications of galactomannans obtained from different commercial and noncommercial sources have been extensively studied over the past decade. Galactomannans show potential in the international trend towards the use of more plant-based products for ecological motives, and their production and application do not cause pollution or disturbance to the ecosystem. Galactomannans are used in a variety of pharmaceutical dosage forms such as tablets, suspensions, hydrogels, and films as an excipient. Besides the simple use as inert excipient, these polysaccharides play role in the modification of drug release, especially in colonic environment, as a matrix or coating material.

*Senna tora* (L.) Roxb. (*S. tora*) belonging to the family Fabaceae is an annual undershrub which grows all over the tropical countries (India, Pakistan, Bangladesh, and west China). It grows well in wasteland as a rainy season weed. It is also known as “Chakramard” in Ayurveda [1, 2].

The* S. tora* seed is composed of hull (27%), endosperm (32%), and germ (41%). Seeds of* S. tora* contain about 23.2% of proteins, rich in all essential amino acids, particularly, methionine and tryptophan. Several compounds belonging to anthraquinone and naphthopyrone groups have been isolated from seeds of this plant. It also contains phenolic glycosides, namely, rubrofusarine triglucoside, nor-rubrofusarin gentiobioside, demethylflavasperone gentiobioside, torachrysone gentiobioside, torachrysone tetraglucoside, and torachrysone apioglucoside. Seed oil contains different percentage of oleic, linoleic, palmitic, stearic, and lignoceric acids [3–5].

Several studies have been conducted throughout the last decade to investigate the biological properties of* S. tora*. Different parts of the plant (Leaves, seed, and root) are reputed for their medicinal value. The leaves of* S. tora* are reported to have antirheumatic activity in folklore practice. Decoction of the leaves is used as laxative. The seeds of* S. tora* have been used in Chinese medicine as aperients, antiasthenic, and diuretic agent. It is also given to improve visual activity (eye diseases) and to treat liver disorders. In Korea, the hot extract of seeds is taken orally for liver protection. Leaves and seeds are used in the treatment of skin disorders (ringworm and itch). Stem bark extract is used for various skin ailments and rheumatic diseases and as laxative. In Ayurveda, the plant is used in “Dadrughani Vati” and “Pamari Taila” [6–10].

The objective of the present research work was extraction of polysaccharide from* Senna tora* L. seeds and further characterization of it as pharmaceutical excipient. Study includes evaluation of micromeritic properties, viscosity and effect of temperature on viscosity, microbial bioburden, and stability and determination of molecular weight of extracted polysaccharide.

## 2. Materials and Methods

### 2.1. Collection of Plant Material

The pods of* S. tora* were collected from Thane district of Maharashtra, India. Plant was authenticated by Dr. Rajendra D. Shinde, Associate Professor, Blatter Herbarium, St. Xavier's College, Mumbai, and was identified as* Senna tora* (L.) Roxb. (herbarium specimen number 8361). The herbarium specimen of* Senna tora* was stored in Ultra College of Pharmacy, Madurai, for future reference. The seeds were separated from pods and stored in cool place till further use.

### 2.2. Isolation and Purification of Gum

The gum was extracted from endosperm using solvent precipitation method as reported in literature [11]. The dried seeds were dehusked and degermed by mechanical treatment followed by milling and screening of the endosperm. The powder was soaked in benzene-ethanol solution (1 : 1) overnight to remove lipids and then it was dried in vacuum oven. The endosperm powder of* S. tora* seeds (10 g) was soaked in 200 mL of distilled water and stirred under overhead stirrer for 3-4 hours. The viscous solution obtained was passed through the muslin. The marc obtained was pressed to remove the mucilage and boiled with 200 mL of water for one hour. Viscous solution obtained was filtered through muslin cloth. The marc obtained was not discarded but it was sent for multiple extractions with decreasing quantity of extracting solvent, that is, water with the increase of number of extractions. The isolation was continued until the material becomes free from mucilage. All the viscous solutions obtained were mixed together. An equal quantity of 10% trichloroacetic acid was added to the mixture to precipitate protein. The solution was centrifuged and the supernatant was precipitated out by addition of acetone in the ratio 1 : 0.5 with continuous stirring. The coagulated mucilage, which formed as a white mass, was transferred to an evaporating dish and dried in vacuum oven at 40°C, powdered, and stored in airtight containers.

### 2.3. Characterization of Gum

The yield of isolated polysaccharide was calculated and organoleptic features were noted. The polysaccharide was evaluated further for the following properties.

### 2.4. Micromeritic Properties

Powder fluidity or flow property is one of the important requirements of pharmaceutical excipient which decides its utility and application in the development of different dosage forms. The flowability of the extracted gum powder was evaluated by determining its angle of repose, bulk, and tapped density using methods reported in the literature [12, 13].

#### 2.4.1. Angle of Repose

The flow characteristics were measured by angle of repose. Improper flow of powder is due to frictional forces between the particles. These frictional forces are quantified by angle of repose. It can be calculated by the following formula:

(1) tan⁡θ=hror  θ=tan−1⁡hr,

where *h* is the height of pile, *r* is the radius of the base of the pile, and *θ* is the angle of repose.

A dry and clean funnel was fixed on to a burette stand at particular height (2-3 cm). A graph paper was placed on the flat surface and a sufficient quantity of the powder (10 g) was allowed to flow slowly through the funnel until the heap touched the tip of the funnel. The circumference of the heap was drawn and the midpoint was located and its radius was measured. The experiment was repeated thrice and the average height and radius were calculated. Using these readings and the above formula, the angle of repose was calculated.

#### 2.4.2. Bulk Density and Tapped Density

A 10 g quantity of gum powder was placed in a 50 mL measuring cylinder and the volume occupied by gum powder without tapping was noted. After 100 taps, the occupied volume was read. The bulk and tap densities were calculated as the ratio of weight to volume.

#### 2.4.3. Hausner's Ratio and Carr Index

Hausner's ratio was calculated as the ratio of tapped density to bulk density of the samples.

*Carr index* (compressibility index) was calculated using the following formula:

(2) % Compressibility =Tapped density−bulk  densityTappeddensity×100.

### 2.5. Viscosity Measurement

The viscosity of 1% w/v solution of* S. tora* gum was measured by Brookfield viscometer at 50 RPM using spindle number 5.

### 2.6. Molecular Weight Determination by Intrinsic Viscosity Method

Viscosity measurements were carried out with Ostwald viscometer tubes after 24 hours of hydration of the gum. Fifty milligrams of the gum was dissolved in 100 mL of water to get concentration of 0.05% w/v. The prepared solution was agitated vigorously for approximately 15 min until the solution become viscous and homogeneous.

The relative viscosity of* S. tora* gum dissolved in distilled water (0.01–0.05 g/dL) was measured using Ostwald viscometer at room temperature. Average molecular weight, *M*_*v*_, was calculated using the Mark-Houwink relationship [Equation (3)] given by Doublier and Launay (1981) for guar gum as modified by Gaisford, Harding, Mitchell, and Bradley (1986) to take into account the different values of mannose (M)/galactose (G) of the galactomannans [14]. Consider

(3) $$\begin{array}{c} \eta =11.55\times 10^{-6}{\left[\;\left(\;1-\alpha \;\right)\times M_{v}\;\right]}^{0.98}, \end{array}$$

where *α* = 1/[(M/G) + 1] and [*η*] is expressed in dL/g.

### 2.7. Effect of Temperature on Viscosity of* S. tora* Gum

Effect of temperature on the viscosity of dilute solution (0.1% w/v in water) of* S. tora* gum was studied experimentally with increase in temperature from 36°C to 100°C. The viscosity measurements were performed using Ostwald viscometer.

### 2.8. Total Microbial Load

The total microbial load is an important parameter which decides the suitability of a substance for use as excipient in pharmaceutical dosage forms. According to many Pharmacopoeias, for synthetic and semisynthetic substances, the total aerobic count should not be more than 100 colony forming units (cfu) per gram, and the total fungal count (including yeasts and molds) should not exceed 50 cfu/g. In the case of excipients from natural origin, the total aerobic count should not be more than 1000 cfu/g and total fungal count should not exceed 100 cfu/g. The total microbial load of the isolated gum sample was determined by plate count method as per Indian Pharmacopoeia [15].

### 2.9. Tests for Presence of Specific Microorganisms

Materials to be used as excipients in dosage forms need to be free from* Escherichia coli*,* Salmonella typhi*,* Pseudomonas aeruginosa*, and* Staphylococcus aureus*, according to Pharmacopoeias. International Conference for Harmonization (ICH) has also supported this criterion. The tests for these microorganisms were carried out as described in Indian Pharmacopoeia.

### 2.10. Accelerated Stability Study

*S. tora* seed polysaccharide was subjected to accelerated stability studies for six months according to ICH guidelines to predict the stability of* S. tora* seed polysaccharide [16]. The samples were analyzed at regular intervals as per the stability protocol (Table 1).

## 3. Result and Discussion

**Figure 1:**
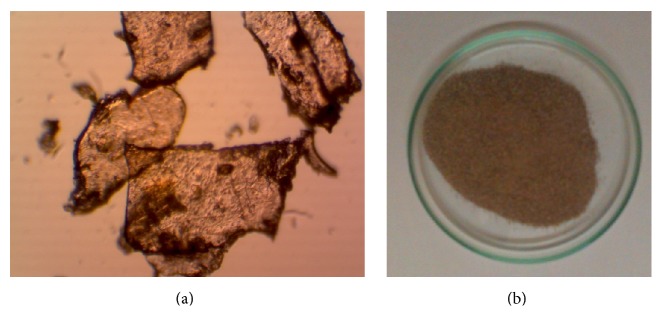
Photograph of gum powder taken (a) under high magnification microscope and (b) using digital camera.

The gum obtained from* S. tora* seeds was an amorphous free flowing odourless powder with dull brown colour (yield = 35% w/w). The photograph of the gum powder was taken under high intensity electron light microscope and using digital camera as depicted in Figures 1(a) and 1(b).

The results of micromeritic study are summarised in Table 2. The bulk density, tapped density, and angle of repose data reveal that* S. tora* gum possesses good flow property. Carr index is frequently used in pharmaceutics as an indication of the compressibility and flowability of a powder. In a free flowing powder, the bulk density and tapped density would be close in value; therefore, Carr index would be small. A Carr index greater than 25 is considered to be an indication of poor flowability and a Carr index below 15 is considered to be of good flowability.

**Figure 2:**
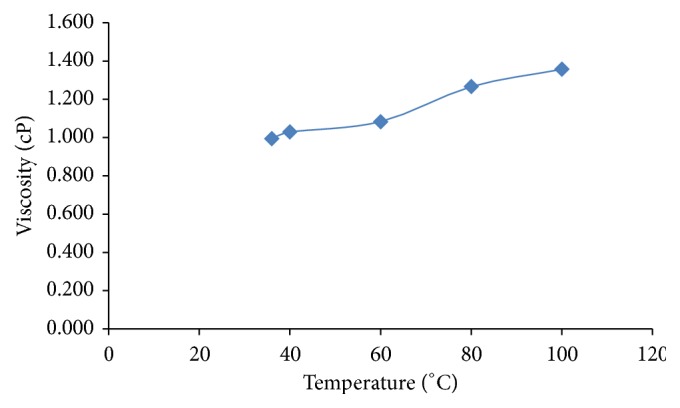
Effect of temperature on viscosity of* S. tora* gum.

Viscosity is the main parameter to assess the quality of natural gums. The applications of any natural gum are dependent on its viscosity. For any polymer to be used in slow release hydrophilic matrix systems, it should possess certain characteristics like fast hydration of the polymer and high gel strength and should be stable during the shelf life of the product. Viscosity of 1% w/v solution of polysaccharide in water was found to be 4520 cP at 50 revolutions per minute (rpm). The results indicated that the polysaccharide isolated from* S. tora* seeds possesses high viscosity with good swelling capacity.* S. tora* seed polysaccharide hydrates quickly and swells rapidly and forms a thick viscous gel around it. Such a high viscosity indicates its utility in the development of various modified release pharmaceutical dosage forms. The effect of temperature on viscosity of* S. tora* gum is depicted in Figure 2.

It has been reported in previous literature that* S. tora* gum contains galactomannans [11]. Galactomannans are formed by several fractions of polymers with different mannose/galactose ratios. At low temperature, molecules with low molecular weight and low mannose/galactose ratio are mainly dissolved. At high temperatures, galactomannan fractions with high molecular weight, greater mannose to galactose relation, and therefore zones without galactose radicals are solved [17]. The results indicated that viscosity of gum solution increases with increase in temperature. This may be due to increase in solubility of galactomannans at higher temperature.

**Figure 3:**
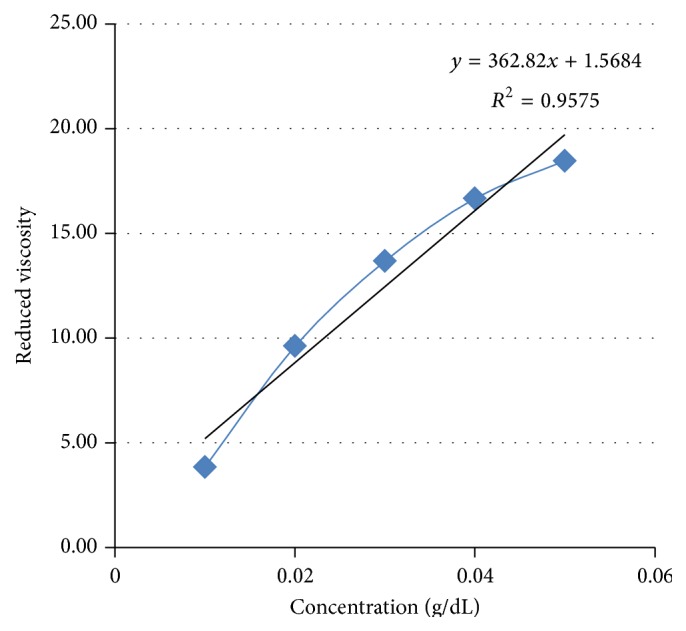
Huggins plot.

The intrinsic viscosity was determined graphically from Huggins plot (reduced viscosity versus concentration graph extrapolated to zero). Huggins plot is represented in Figure 3.

The intrinsic viscosity obtained was 1.568 dL/g. The average molecular weight of purified* S. tora* gum was found to be 198 kDa by intrinsic viscosity method.

**Figure 4:**
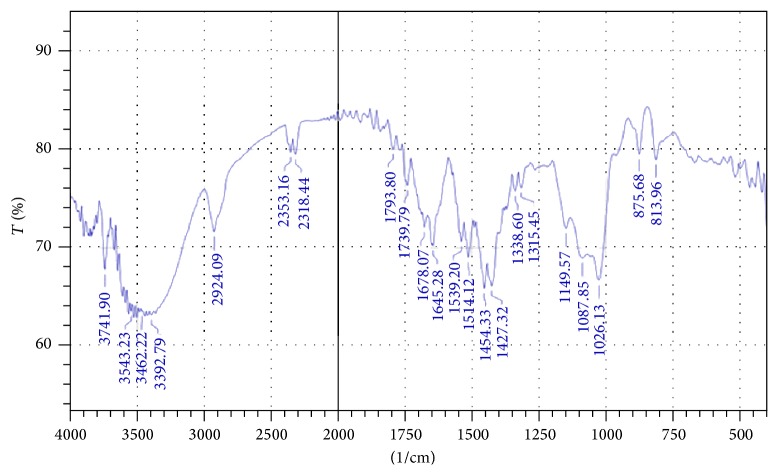
FTIR spectrum of the* S. tora* polysaccharide (initial sample).

Figure 4 represents FTIR spectrum of* S. tora* polysaccharide.

The FTIR spectrum of the initial sample (Figure 4) of isolated polysaccharide confirmed the presence of galactomannans. The interpretation was done using previously published literatures [18–23]. The FTIR data of the polysaccharide is presented in Table 3.

The total microbial load was found to be 340.23 ± 10.79 cfu/g of bacteria and 44.39 ± 9.31 cfu/g of fungi. The total microbial load of the polysaccharide was thus within the acceptable limits (the acceptable limit for total microbial count is 1000 cfu/g of bacteria and 100 cfu/g of fungi for natural products). In specific tests for microorganisms, it was found that* Escherichia coli*,* Salmonella typhi*,* Pseudomonas aeruginosa*, and* Staphylococcus aureus* were absent, which is essential criteria for their use as excipients in dosage forms.

**Figure 5:**
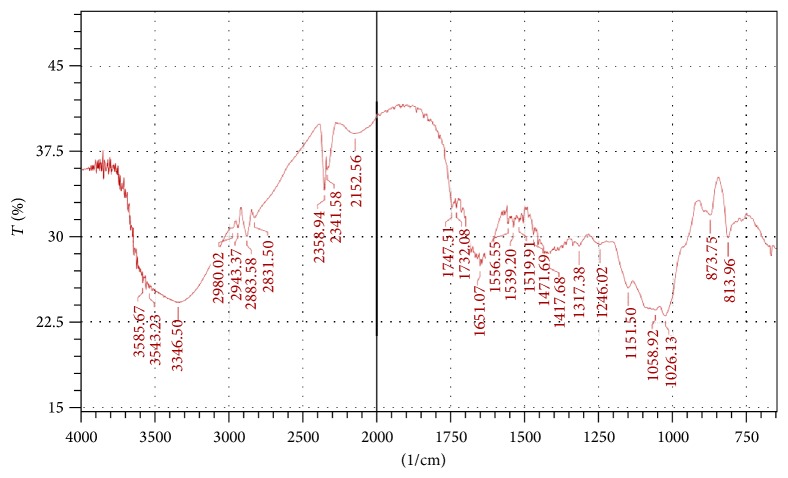
FTIR spectrum of the* S. tora* polysaccharide (after 6 month).

The results of accelerated stability studies on* S. tora* seed polysaccharide powder showed that there is no significant difference between the initial and final samples withdrawn at time interval 6 months at 40°C/75% RH. Slight changes in pH and moisture content values were observed. The studies proved that the* S. tora* seed polysaccharide is stable for a long period of time. FTIR study revealed the absence of degradation or decomposition of polysaccharide at accelerated stability conditions for six months as shown in Figure 5.

## 4. Conclusion

The polysaccharide (gum) extracted from* S. tora* seeds possesses good flow property. The gum possesses excellent viscosity. The gum polysaccharide was found to be stable at accelerated stability condition. Microbial studies confirmed its suitability as an excipient. Based on the physicochemical, microbiological characteristics and stability studies, it may be pointed out that the polysaccharide isolated from* S. tora* seeds has the required properties and could be used as an excipient for pharmaceutical dosage forms.

Thus there is need to investigate further* S. tora* polysaccharide as an excipient in different pharmaceutical dosage forms. It may provide an alternative to synthetic or semisynthetic excipients/polymers currently used in the pharmaceutical industry.

## Figures and Tables

**Table 1:** Accelerated stability protocol of *S. tora* gum.

*Product*:* S. tora* seed polysaccharide		
Quantity loaded: 20 g approx. in each Petri dish		
Quantity Sample: 2 g approx.		

Sampling interval	Storage conditions	Test

Initial First month Second month Third month Sixth month	Temperature = 40°C Humidity = 75% RH	(1) Organoleptic evaluation (2) pH of 1% w/v solution (3) Moisture content (4) Microbial count (5) FTIR

**Table 2:** Results of micromeritic properties of *S. tora* seed gum.

Parameters	Results^*∗*^
Bulk density (g/mL)	0.51 ± 0.047
Tapped density (g/mL)	0.60 ± 0.039
Hausner's ratio	1.17
Carr index	14.29
Angle of repose	29°44′ ± 0.032

^*∗*^The values represent mean ± SD (*n* = 3).

**Table 3:** Interpretation of FTIR data of polysaccharide.

Wave length	Peak assignments
813 and 875 cm^−1^	Anomeric configurations (*α* and *β* conformers) and glycosidic linkages
1198 and 983 cm^−1^	Stretching vibration of C–O in C–O–H bonds
1149 cm^−1^	Bending vibrational modes of C–O, present in the pyranose ring
1134 and 983 cm^−1^	C–OH bending
2800–3000 cm^−1^	C–H stretching
3100–3500 cm^−1^	O–H stretching vibration
